# Her Heart's Mistake

**Published:** 1889-09-28

**Authors:** E. D. Cumming

**Affiliations:** Author of "An Ordeal by Silence


### Her Heart's Mistake.

By E. D. Cumming, Author of "An Ordeal by Silence.

*(Concluded from page 397.)*

CHAPTER XXV.—A Night in Camp.

"Well, Bairdsley, what have you been towelling him for this evening?" enquired Major Carrick, of the 69th Punjaubees, indicating a Pathan camel-driver who was rubbing his bare shoulders, and muttering deep curses below his breath.

"Oh, he's been playing the fool again, as usual. How he could have managed it I don't know, but he has succeeded in smashing my camp-chair this march."

Bairdsley pointed to a dilapidated folding chair, whose remains lay with other goods which had just been removed from the kneeling camel.

"It looks strong enough to stand ordinary wear and tear. That's been done by way of retaliation for the licking you gave him yesterday."

"So I supposed, and inflicted punishment accordingly. We'll see who wins before long. I'll give him a double dose next time he breaks anything."

"You're not dealing with the mild Hindoo, my dear fellow. Better be careful how far you go with a semi-savage like that. It's not safe to drive a Pathan to extremities up here."

"Up here" was a valley in the bare inhospitable mountain ranges north-west of Peshawur, where the mixed column to which Bairdsley was attached had halted for the night. It was a wide amphitheatre of grass land, hemmed in on every side by bold ridges and walls of naked rock. A swiftly flowing stream ran through it, entering by a narrow canon which was visible from the spot chosen for the encampment; but where it made its exit to join the Cabul river could not be detected. The valley was an oasis in the midst of barren mountains, but there was no sign of human habitation in it.

The punitive expedition, consisting of three companies of the 110th, the 69th Punjaub infantry, and three mountain guns carried on elephants, had just completed its eighth day's march from Peshawur. The officer commanding had pushed on as fast as possible, and confidently hoped that the next day would bring his force within sight of the hill fortress which was the enemy's stronghold, and whither the audacious raiders had retreated before the advancing columns.

They were known to be close at hand, for, though they had never allowed themselves to be seen, three or four sepoys had been wounded and a number of camels killed or disabled by jazil fire from seemingly inaccessible crags overlooking the line of march. The animals thus lost had been replaced by others, picked up at villages passed on the route, whose owners would murder the Feringhi or accept his rupees with equal complacency. The camel that carried Bairdsley's private effects happened to be one of the first victims, and as its load comprised his bedding, spare kit, and boots, he was glad to engage the first man who presented himself with a suitable beast. He was a tall, muscular young Pathan, whose language was unintelligible to all but the guides; and he did his work with a cool indifference to his employer's orders that irritated Bairdsley intensely. To do the latter justice, he had instructed him carefully, through an interpreter, how to secure the many shaped packages safely upon his camel; but every evening when that animal knelt down to be unladen before Bairdsley's tent, some trifle was sure to be missing or broken. And the rod was called into requisition, and plied vigorously in spite of vows of vengeance, which were unmistakable in their purport.

It was useless to remonstrate with the man after his first thrashing, and every morning a dialogue on the following lines might have been heard between Bairdsley and the Pathan:

Bairdsley (speaking in broken Hindustani): "Take that bag off, and tie it *there!*"

Camel-driver (in his own dialect): "It is safe there!"

Bairdsley: "My order is, tie it there!!"

Camel-driver: "It will cut her skin!!"

Bairdsley: "Do what you are told!!!"

Camel-driver: "I won't hurt my camel for you!!!"

Eventually, Bairdsley would thrust the man aside and, regardless of his protests, make the desired alteration himself; the invariable consequence being a loss or breakage of some description, which the Pathan attributed to the white man's ignorance of camel lading, and which was punished by a flogging more or less severe.

That day's march, like many a previous one, had lain over high-lands, where the atmosphere was tolerably cool; but down in the valley the heat was overpowering. The evening was close and oppressive, and, though the thermometer was not abnormally high, the dampness of the situation made the temperature more trying than the dry heat to which dwellers in the Indian plains are innured.

Bairdsley watched the men pitch his tent and move his furniture into it with a keen eye to the discovery of further damages. He found no more cause for complaint, however, and limped inside to enjoy a wash. He had galled one of his feet slightly, and was very tired besides. He promised himself an early retirement to bed and a good long sleep that night. They would come to the end of these wearisome marches to-morrow and see a little work, which was something to be thankful for.

"And, by Jove! as soon as we get back to headquarters I'll send in my papers and cut the service," he said to himself; "I'm sick to death of the business."

But, as this fairly indicates the frame of mind in which Bairdsley looked back upon each day's labours, it meant very little; he was, in fact, all anxiety to come up with the enemy and see his first fight.

He changed his dusty, travel-stained uniform, and strolled over to the mess-tent, where the officers belonging to the force were assembling for dinner. Daylight was rapidly dying away, and the long table was illuminated with lamps and candles, the latter looking limp and dejected, owing to the influence of the heat.

All around stood the dirty-white canvas bell tents of the troops, the sepoys hard at work, each man tending a brass lotah of boiling rice over his own tiny fire. On the British side of the camp the men were lounging about in various stages of undress, smoking and talking in groups or watching the cooks prepare the evening meal. Many were bathing in the stream and dressing on its bank, while the unfortunates told off on "sentry-go" tramped to and fro upon their beats, eyeing them enviously. The sun had disappeared behind the grim mountain peaks in the west, and darkness was gathering when dinner was announced to the hungry officers waiting about the marquee.

"Well, to-morrow brings the end of this pleasure trip, Bairdsley," said that gentleman's neighbour as they sat down to table.

"I can't say I'm sorry for it."

"Nor am I; the tramp back will be a worse one though, with wounded to carry."

"They say this hill fort is a nasty one to get at," remarked Bairdsley.

"No one has ever seen it, not even the Intelligence Department; the guides want the C.O. to believe it's impregnable, but Bainbridge is hard to convince."

"Well, we shall see to-morrow," replied Bairdsley, smothering a yawn, "I am dead sleepy to-night."

"This valley is like a cauldron; all the heat seems to collect in it. I'd put mybed outside if I wasn't afraid of fever."

"I thought of doing the same."

"You'd better not if you're a feverish subject; even canvas is some protection from malaria, but I shouldn't care to stay here long."

"It's a lovely spot; one would hardly expect to find such an Eden amongst these forbidding-looking rocks."

"An excellent spot for a cemetery," returned the other cynically, "but hardly good enough for the living man."

"I was surprised to see it so completely uninhabited."

"The hill man naturally prefers a mountain top, when he lives by harrying his neighbours."

Few of the men were inclined to dawdle over their dinner that evening, and they dropped off one by one to seek their beds. Bairdsley was one of the first to go, and after he withdrew, Major Carrick referred to the rash cruelty with which he had treated the Pathan camel-driver.

"He don't understand the nature of these fellows. If he was under my command I'd put a stop to his pranks as much for his own sake as the unlucky nigger."

"Why, do you think the man will turn on him one of these days?"

"He's not likely to do it openly, but if he gets a favourable opportunity of doing the job he'll stick that nasty knife he carries between Bairdsley's ribs."

They had risen from the table, and were now smoking their cigars in front of the marquee. The camp was in perfect silence except for their own voices.

"He looked a perfect fiend when he led away his camel," remarked another officer; "I don't fancy he will take many more lickings quietly."

"I'm quite sure of it," said Major Carrick, emphatically. "I know these fellows well. Where is Bairdsley's tent, Thomson—do you know?"

"Close to that pile of commissariat stores. Why do you ask?"

"I was thinking that he would be wise to take precautions to-night."

"Oh, he'll be all right. There's a sentry over the stores, within a dozen yards of him."

"The moon will be up in another hour or so, and it will be as bright as day. No one could get near his tent without being seen," said another man.

Major Carrick, with his knowledge of the hill tribesmen, was not altogether satisfied of that, but said nothing further; he was not a croaker, and, after all, it wasn't likely that the Pathan would attempt mischief almost under the eyes of an armed sentry. He threw away his cheroot, and bidding the others good-night, went off to turn in himself.

Bairdsley, having exchanged his clothes for pyjamas, threw open the flaps of his tent-door and tied them back, to admit as much air as possible; having done so, he stood for a few moments looking out into the starlight, yawned widely, and came inside. His revolver was hanging with his sword on the pole, and as he passed it he hesitated; he was not a nervous man, but he reflected that that blackguard did look very ugly when he had received his thrashing, and might take it into his head to pay him a nocturnal visit. It could do no harm to be ready for him, so he drew the pistol from its holster and turned the chambers to feel the tips of the bullets; all six were loaded, so, with another yawn, he thrust the weapon under his pillow and turned out his lamp.

Tired as he was, he could not go to sleep at once. His wife's face rose before him, and their last parting came vividly into his mind. He had treated her as few men had ever treated a woman before, and he could never dare face her again unless she called him to her. The discovery of Anderson's letter had given her the upper hand, and he could think of no way by which to reinstate himself in her good graces. He had accused her of keeping up a secret correspondence with that barrister, too, knowing the charge to be as foundationless as cruel. He was bitterly sorry for it now. She had deserved better treatment at his hands. She had never failed in her duty to him as a wife, and when he got back to Peshawur he would write and make what reparation he could by letter.

Phew! how hot the night was, and how pitchy dark. He turned over and lay upon his back, throwing off the sheet which covered him as he did so, and fell thinking of Kate again. What had brought him to see his conduct towards her in colours so black to-night? He had never felt its baseness, or recognised his own unworthiness of her so keenly before. She had never suspected his true character. It was beyond her ken. She was pure as the angels of heaven. Would to God that he could undo what he had done.

And Bairdsley, with none to see or hear him, whispered the first prayer that had passed his lips for many a year, and presently he fell asleep.

The night wore on, and soon the sentry outside saw above the hills on the other side of the valley the faint light in the sky, which heralded the rising of the moon. And another watcher, peering between the bags and boxes amongst which he had patiently lain hid since dusk, saw it too. From his lair in the heap of stores the Pathan, who bore the marks of Bairdsley's cane across his shoulders, drew himself silently, warily, as a beast of prey. When he had half emerged from his hiding-place he stopped and looked round. He wore no turban nor other garment than a cloth folded round his loins. Holding his breath, he looked for the sentry. He could see his figure against the sky-line; the man was leaning on his rifle as he watched the moonrise; his back was turned to the goods he guarded, and he was humming a tune softly to himself. The Pathan drew himself clear of the hole, felt for something in his waistcloth, and lying almost prone upon his belly, began to crawl noiselessly as a snake towards the open door of Bairdsley's tent. He had seen his oppressor's every movement since he entered it, and had been waiting until he should be asleep. He crept along, keeping his head turned to watch the sentry's movements, and feeling before him to avoid tent-pegs and ropes in his advance. The soldier resumed his monotonous tramp, and the instant he moved, the Pathan sank down flat upon the grass, still as a corpse, but keeping his lynx eyes upon him. The sentry's beat took him past the heap of commissariat goods, and as he disappeared behind it, the creeping figure worked its way a little nearer the tent-door. In a few moments he heard the sound of returning footsteps, and lay motionless again, until the sentry was once more out of sight. And thus writhing and crouching by turns, he gradually crossed the open space between his hiding-place and his victim.

He had reached the tent-door now, and drew himself up to a kneeling position to listen; the steady regular breathing of the Feringhi proved that he was asleep, and the Pathan drew a long knife from his waistcloth, and took the blade between his teeth, leaving his hands free to feel his way into the tent. He could lose no time. Already the silver disc of the moon was showing its rim above the mountains, and delay would be dangerous. He dropped on all fours and crept stealthily over to the bed, where he once more knelt upright at the sleeper's side. He took the knife from his mouth, and raising it in his right hand, felt with light fingers over the prostrate form for the spot where he should strike. Then, for a moment, he paused to enjoy the sweetness of his revenge. Bairdsley stirred in his sleep. The assassin clenched his teeth, and, with a downward stroke, swift as a lightning flash, drove the dagger into his breast up to the hilt. There was a low gurgling sob, and the Pathan knew that his blow had done its work.

Long before day broke he was safe in the mountain retreat of his clan, thirty miles away from the scene of his crime.

### Chapter XXVI.—Conclusion.

"You never heard particulars about his death, did you, Kate?"

"No father; I thought some of the officers who were with him would have written to me about it, but they did not. Colonel Collins could only give me the information he received by telegram from Peshawur a fortnight after it happened, when the troops had returned."

The tragic truth of Bairdsley's murder had been carefully concealed from his wife by the officer who broke the news of his death to her. He had given her to understand that her husband had fallen, as many another man had done, in the attack upon that nameless hill fort in the mountains, and the actual facts of the case had not been blown to her by any side wind.

As soon as the intelligence reached her, she left Simla, and returned to Delhi, where she made arrangements for the sale of furniture, etc., and collected the property she had to take home. Then she went down to Bombay, caught the P. and O. steamer "Rome" on the point of sailing, and took her passage in it, turning her back for ever upon the land where she had suffered so much. She was now living with her father in Arran, whither he had taken her for quietness and rest immediately on her return. They occupied a little cottage overlooking Sannox Bay, a secluded spot, where few came to intrude upon their privacy, and where they could spend the long warm August days as they pleased, without fear of interruption. This evening they had just returned from a ramble over the hills behind the cottage, and were sitting on a bench in the little garden. It was the first time that the Doctor had asked his daughter so straightforward a question about her husband's death. He shrank from touching upon the subject, though he knew well the feelings with which she regarded the dead man. Regret could have little place among them, for the tie which death had severed had been wearing out her life.

"It was strange that you should have known so little of this Miss Carsworth, father," said Kate, who avoided all reference to her late husband as far as she could. She wore mourning for him, but was too transparently honest to simulate grief she did not feel.

"She was my father's cousin, and the uncle who left the property cut him off in her favour. I never knew the exact circumstances, but there was some talk of legal proceedings which estranged them, and all communication had ceased between them when I was a lad. I never saw her, and knew nothing about her or her intentions until I received your letter."

"And you have never attempted to discover her whereabouts?"

"No; I don't see that it would answer any good purpose."

"Do you not think that you ought to make yourself known to her?" asked Kate.

"Why should I do so, my dear? She knows that she can find me out, if she has a mind to, through the War Office. I should have no motive in seeking her acquaintance except for her money."

"She is a very old woman. I wonder if she lives alone." The Doctor smiled. "The rich are never lonely except by their own choice, Kate."

They often discussed Miss Carsworth and her will, but nothing would induce Dr. Harrison to take any steps towards seeking her out, though his daughter had pressed it upon him as a duty he owed to himself. He would not move in the matter, he said; if his relative had any wish to find him, she knew how to set about it, and he would wait her pleasure.

Had he only known it, Miss Carsworth had watched Kate's career through Mr. Anderson, giving that gentleman strict injunctions to keep the provisions of her will secret from the parties interested. But her man of business had so far betrayed his trust as to impart his knowledge to his friend and schoolfellow, George Bairdsley, in return for services rendered when he, Mr. Robert Anderson, was in serious money difficulties. He had told him jocularly that he could give him the address of an heiress who did not know her own value, if he liked; and Bairdsley in the same spirit had accepted the information, at first without the remotest idea of making use of it. But before many months had passed, he began to think that fortune hunting in India would be an agreeable change from the monotony of barrack life in England; and learning from Anderson that the Harrisons were at Meerut, where the second battalion of his own regiment was stationed, embarked upon the enterprise in sober earnest.

The old lady had been kept regularly informed of her heiress's doings, and had been made aware of Captain Bairdsley's death; but she did not know that Kate had actually returned from the East. She was a somewhat whimsical personage, and had no great curiosity to see the girl to whom she had bequeathed her money.

"I mightn't like her you know, Peek," she said to her companion, "and if I didn't like her I should go leaving my money to you, or the Home for Lost Dogs, or the Hospital for Incurables, or doing something foolish with it. Don't you think so?"

And Mrs. Peek would have gladly taken her chance with the incurables and lost dogs, but knew the duties of a lady's companion, and agreed with her employer.

"If I met her by chance I could judge of her character, as she doesn't know that I have even mentioned her name in my will, and I don't mean that she shall until the day comes for her to take the money; it can't be very far away now."

"Oh, you mustn't say that, you mustn't think that," responded Mrs. Peek.

"But I do say that, Peek; I'm an old woman, and I can't live for ever."

Mrs. Peek applied her handkerchief to her eyes, and murmured, "No, no," with tender softness.

"Don't be a fool, Peek. I can think of these things calmly, so why can't you?"

But Mrs. Peek declined to be comforted; she, poor soul, had had a hard fight with the world until Miss Carsworth appointed her to the post she now occupied, and that lady's death would turn her adrift again, with a thousand pound legacy and her little savings. They were not altogether companion's tears.

"It's a sad pity she has lost her husband. (Do stop crying, Peek!) A fine, handsome man he was, Anderson tells me. 'Twould have been a grand surprise for a young couple. Now, I'm always afraid that she may fall a prey to some adventurer as soon as I'm gone."

Miss Carsworth sighed as she spoke. She did not know already what expectations had done for Kate.

"Dr. Harrison is alive, isn't he?" asked Mrs. Peek, after a pause.

"Yes, he's at Cheltenham. I don't know him, and he has never taken the trouble to look for me since he came home from India."

"Why not leave your money to him, to go to his daughter if she marries again with his approval, Miss Carsworth?"

"That's the most sensible thing you have said for a month, Peek. I'll think about it."

And Miss Carsworth did think about it; she thought about it for three days, and finally directed Mrs. Peek to telegraph Mr. Anderson to come down to Southsea at once and bring her will with him. The alteration was duly made, and the old lady told her companion that now she could die with an easy conscience. Dr. Harrison was to inherit her wealth, enjoying the income until Kate married with his sanction, when it was to be settled upon her.

"It's rather hard lines upon him, Peek. But I owe him one for neglecting me; he ought to have paid his only relation some attention, distant as the connection is."

"Of course he ought," acquiesced Mrs. Peek.

And while these things were being enacted in the South, Dr. Harrison and his daughter were quietly enjoying themselves in their island retreat in the North, ignorant of the fact that Miss Carsworth marvelled at their silence.

Warren's name had not been mentioned by either, though he was ever present in the thoughts of both. The Doctor heard from him occasionally, but he never showed his letters to Kate, nor told her when he received them. She never referred to him; she did not even know whether he had heard of her return to England. He must, of course, have seen the report of Bairdsley's death in the papers, and might guess the natural sequence of events to which the disaster would give rise.

September came, and the autumnal rain set in on the West Coast, nowhere more diligently than in Arran. Goatfell and his brethren were shrouded in everlasting mist, and the rocky streams swelled into roaring torrents, which flooded the roads and carried away the light bridges which spanned them. The rooms in the cottage which the Harrisons rented were small and confined, and after a week's wet weather the Doctor began to talk of making a move.

"It's no good staying here, Kate," he would say as he came in to breakfast every morning. "We can't go out and can't do anything in the house. If it doesn't clear up in a day or two I shall turn my head south again."

But the heavy clouds hung over the island as though loth to leave it, and it seemed that the short summer had indeed come to an end. So Dr. Harrison and his daughter took unto themselves wings, and forsook their sea-girt refuge for the more civilised world of Cheltenham.

The Doctor was at home in that haunt of Anglo-Indians, but Kate found almost as little to do there as in Arran. She read a great deal, spent at least two days a week in answering the shoals of letters of condolence which came from her many friends in India, and filled up the rest of her time in taking long walks alone or with her father. It happened one morning, when she was wending her solitary way along a country lane, that she came upon an old lady leaning over a gate in the hedge, waving an umbrella, and vociferating "Snap! Snap!" at the highest pitch of her voice. She was very red and breathless, and looked so disturbed and anxious when Kate came up that she stopped to enquire whether she could be of any assistance.

"It's Snap," panted the old lady, pointing with her umbrella to a small white fox-terrier which was barking itself hoarse at a cow. "He'll be killed, and there isn't a man to help in sight."

Kate looked over into the field, and took in the situation at a glance. The cow was dancing around with clumsy agility, butting and kicking at her tormentor without any effect but to excite the dog to greater efforts.

"He'll be tossed and killed," said the old lady, in quavering tones; "I feel it; I know it."

Kate Harrison was one of those few young ladies who are not "afraid of cows," and, seeing that the dog's owner was really apprehensive for its safety, she unlatched the gate and made for the cow without hesitation. The old lady's excitement and distress increased as she advanced into the field, and she shouted in turn warnings and orders to come back, until she had to cease for lack of breath.

It was a very easy task that Kate had undertaken; the cow only wanted to be left alone, and seemed to understand the nature of her mission. Snap had had quite as much fun as his corpulence allowed him to enjoy without danger of apoplexy, and three minutes after Kate had passed into the field the cow was trotting quietly in one direction, and the dog, with his tongue hanging out of his mouth, was sitting at his mistress's side awaiting his rescuer's return.

The old lady's gratitude was so voluble and sincere that Kate could not escape from her without appearing rude; and she joined her in her walk back towards Cheltenham, listening with rather indifferent attention to a long biographical sketch of the audacious Snap, from his puppyhood until the present time.

"I left my companion at home to-day," said the old lady, as she stopped before a neat little house with a well-kept garden before it; "I'll never take that wretch out again unless I have her with me though—never!

Kate's dress had attracted her attention now, and under pretext of looking at her watch, she put on her spectacles to see it better.

"I wish I'd asked her name," she said to herself as she entered her gate; "such a young creature to be in widow's weeds."

She paused as she reached the door, and struck her umbrella on the step.

"What a dolt you are, Mary Carsworth! That must be Mrs. Bairdsley!"

It *was* singular that this surmise had not suggested itself sooner, for she had come to Cheltenham with no other purpose than to see the Harrisons for herself without letting them know of her presence. She had been there for a month, but enquiries made at the post office had revealed that the Doctor and his daughter had gone away to Scotland, and were not expected back before the end of October. Miss Carsworth had, therefore, decided to wait until they returned, but was unprepared to see them so soon as this.

Kate met the old lady whose dog she had rescued two or three times before she discovered who she was. She answered many questions about herself, and the old lady was soon in possession of the story of her life in India, her marriage, her lost child, and her husband's death; but only on the fourth occasion of their meeting did she learn her new friend's name. And when Miss Carsworth told it her she did so with matter-of-fact coolness.

"I'm a distant connection of your father's, my dear, but I don't even know him by sight. You might bring him to see me one afternoon when you have nothing better to do. I'm always at home after five."

The Doctor looked puzzled when his daughter brought the message. He knew the house Miss Carsworth had taken, which was by no means one which a woman of her wealth would have selected unless she intended to keep her real means secret.

"I shouldn't be surprised if she came here on purpose to find us out," he said, thrusting his hands into his pockets and going to the window. "However, we will accept her invitation to go and see her."

So a few days afterwards Dr. Harrison and Kate made the first of what initiated a long series of friendly visits. Miss Carsworth liked them both, and deplored to Mrs. Peek the misfortune it was that she had not known them long ago.

"If Kate marries in my lifetime, I'll have to alter that will again," she said one evening to Mrs. Peek. "She's too good a girl to be left single long."

December came, and Miss Carsworth put forward a scheme she had been cherishing for a month past. The Doctor and Kate must come with her to Nice and spend the winter there; she had her own villa, and it was frightfully dull, alone with Peek. They must come; she would take no denial, for they had nothing to keep them in England. They agreed, after much pressure, and spent a week in London on the way. There Kate once more saw Richard Warren. He had changed very little in appearance, and not at all in other respects. He said no word about the past, and nothing of the hopes he still faithfully nursed for the future; but when she bade him good-bye they knew well that they were to meet again.

And they did six months afterwards.

